# Supplementary material for: AI-based histopathology image analysis reveals a distinct subset of endometrial cancers
Source: Nat Commun. 2024 Jun 26;15:4973. doi: 10.1038/s41467-024-49017-2 (PMC11208496; doi:10.1038/s41467-024-49017-2)
Supplement: Supplementary file 1 — Supplementary Information [file 41467_2024_49017_MOESM1_ESM.pdf]

Supplementary Materials

List of Figures

Supplementary Fig. 1: ROC plots of the trained VarMIL networks in the (A) discovery, (B) BC validation, and (C) CC validation sets.

Supplementary Fig. 2: DSS and PFS KM curves associated with NSMP, *p53abn-like NSMP*, and p53abn cases in the (A) discovery set and the (B) BC validation set. The reported p-values compare the significance between p53abn-like NSMP and p53abn, computed using a two-sided log-rank statistical test.

Supplementary Fig. 3: DSS and PFS KM curves associated with *NSMP-like p53abn* and p53abn cases in the (A) discovery set, and the (B) BC validation set. *NSMP-like p53abn* represents cases that are p53abn as assessed by IHC but classified as NSMP based on H&E slides by the AI model (The reported p-values compare the significance between p53abn-like NSMP and p53abn, computed using a two-sided log-rank statistical test).

Supplementary Figure 4: Performance benchmarking of Vanilla (A), IDaRS (B), Histogram (C), DeepMIL (D), VLAD (E), CLAM-MB (F), CLAM-SB (G), and TransMIL(H) models for the discovery and validation sets.

Supplementary Figure 5: Kaplan Meier curves along associated with the p53abn-like NSMP and NSMP groups for the (A) discovery, (B) BC validation, and CC validation cohorts using various deep learning frameworks. Note, DSS was not available for the TCGA part of the discovery cohort (The reported p-values compare the significance between p53abn-like NSMP and p53abn, computed using a two-sided log-rank statistical test).

Supplementary Figure 6: Histopathological features from the slides in the BC validation cohort utilizing (A) Densnet121, (B) Swin, and (C) ResNet50 feature representations from the slides demonstrate that p53abn-like NSMP and the rest of the NSMP cases constitute two separate clusters.

Supplementary Fig. 7: An overview of AI tumor-normal classifier and automatic annotation. (A) Extracting tumor and stroma patches from manually annotated slide and training a binary deep model. Red regions show annotated tumor regions, while green depicts stroma sections. (B) Extracting all patches from un-annotated slides and feeding them to the trained model to highlight only the tumor ones. The final tumor regions are shown by red contours.

List of Tables

Supplementary Table 1: Overview of cohorts.

Supplementary Table 2: Outcome information of the discovery and validation cohorts.

Supplementary Table 3: Treatment information of the discovery and validation cohorts.

Supplementary Table 4: Distribution of samples across different centres in the CC validation cohort.

Supplementary Table 5: Accuracy and other performance measures of the binary tumor-stroma classifier.

Supplementary Table 6: Performance metrics of the deep learning model for p53abn vs. NSMP classifier (based on VarMIL method). The results are based on mean ± std of 10 cross-validation splits.

Supplementary Table 7: Detailed accuracy and other performance measures of the VarMIL network.

Supplementary Table 8: Statistics of NSMP and *p53abn-like NSMP* patients based on p53abn vs. NSMP classifiers.

Supplementary Table 9: Performance benchmarking of Vanilla (A), IDaRS (B), Histogram (C), DeepMIL (D), VLAD (E), CLAM-MB (F), CLAM-SB (G), and TransMIL (H) models for the discovery and validation sets.

Supplementary Table 10: Statistics of NSMP and p53abn-like NSMP patients based on p53abn vs. NSMP Vanilla (A), IDaRS (B), Histogram (C), DeepMIL (D), VLAD (E), CLAM-MB (F), CLAM-SB (G), and TransMIL (H) models for the discovery and validation sets.

Supplementary Table 11: Clinicopathologic features of the *p53abn-like NSMP* group in the discovery set.

Supplementary Table 12: Clinicopathologic features of the *p53abn-like NSMP* group in the BC validation set without excluding any patients.

Supplementary Table 13: Clinicopathologic features of the *p53abn-like NSMP* group in the CC validation set without excluding any patients.

Supplementary Table 14: Multi-variate Cox regression analysis showing the prognostic significance of *p53abn-like NSMP* group PFS for the entire cohort.

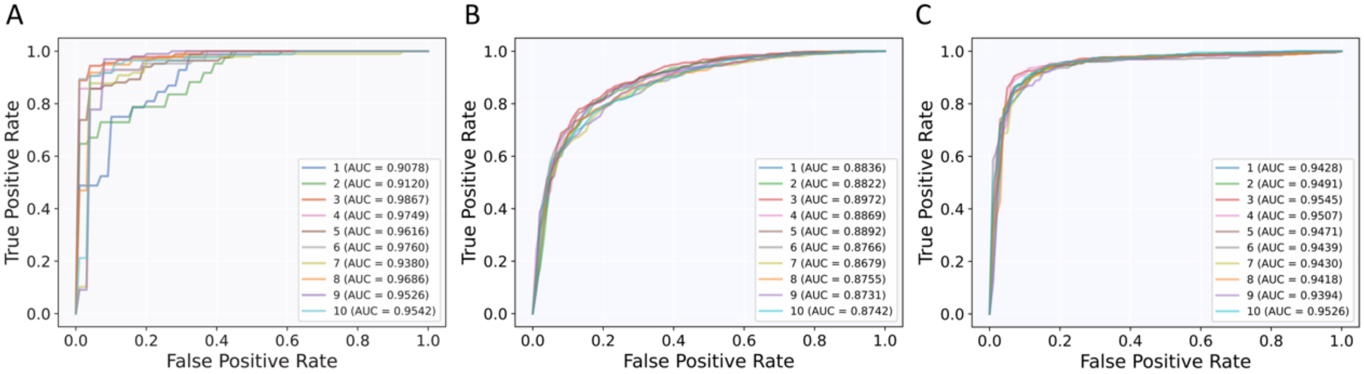

Supplementary Fig. 1: ROC plots of the trained VarMIL networks in the (A) discovery, (B) BC validation, and (C) CC validation sets.

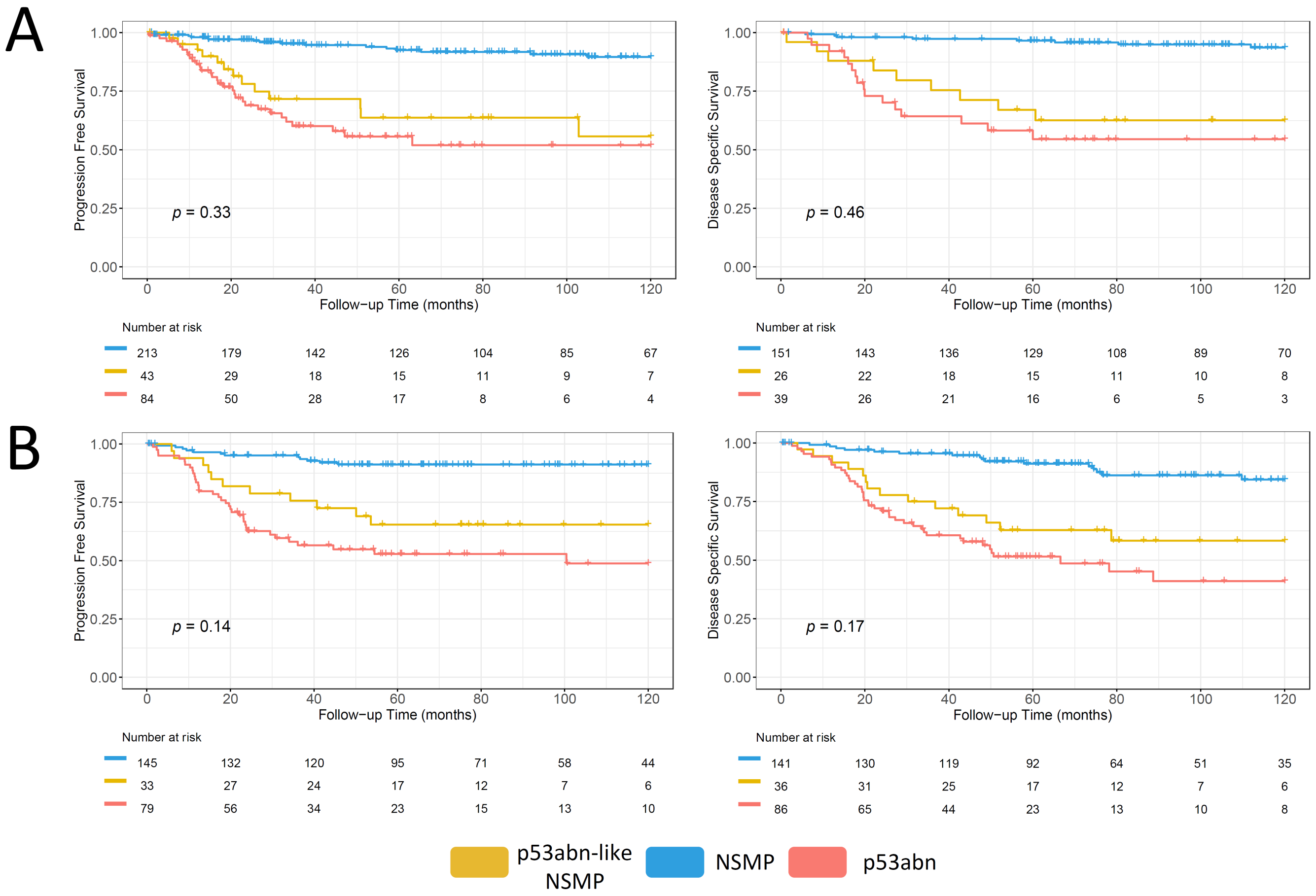

Supplementary Fig. 2: DSS and PFS KM curves associated with NSMP, *p53abn-like NSMP*, and p53abn cases in the (A) discovery set and the (B) BC validation set. The reported p-values compare the significance between p53abn-like NSMP and p53abn, computed using a two-sided log-rank statistical test.

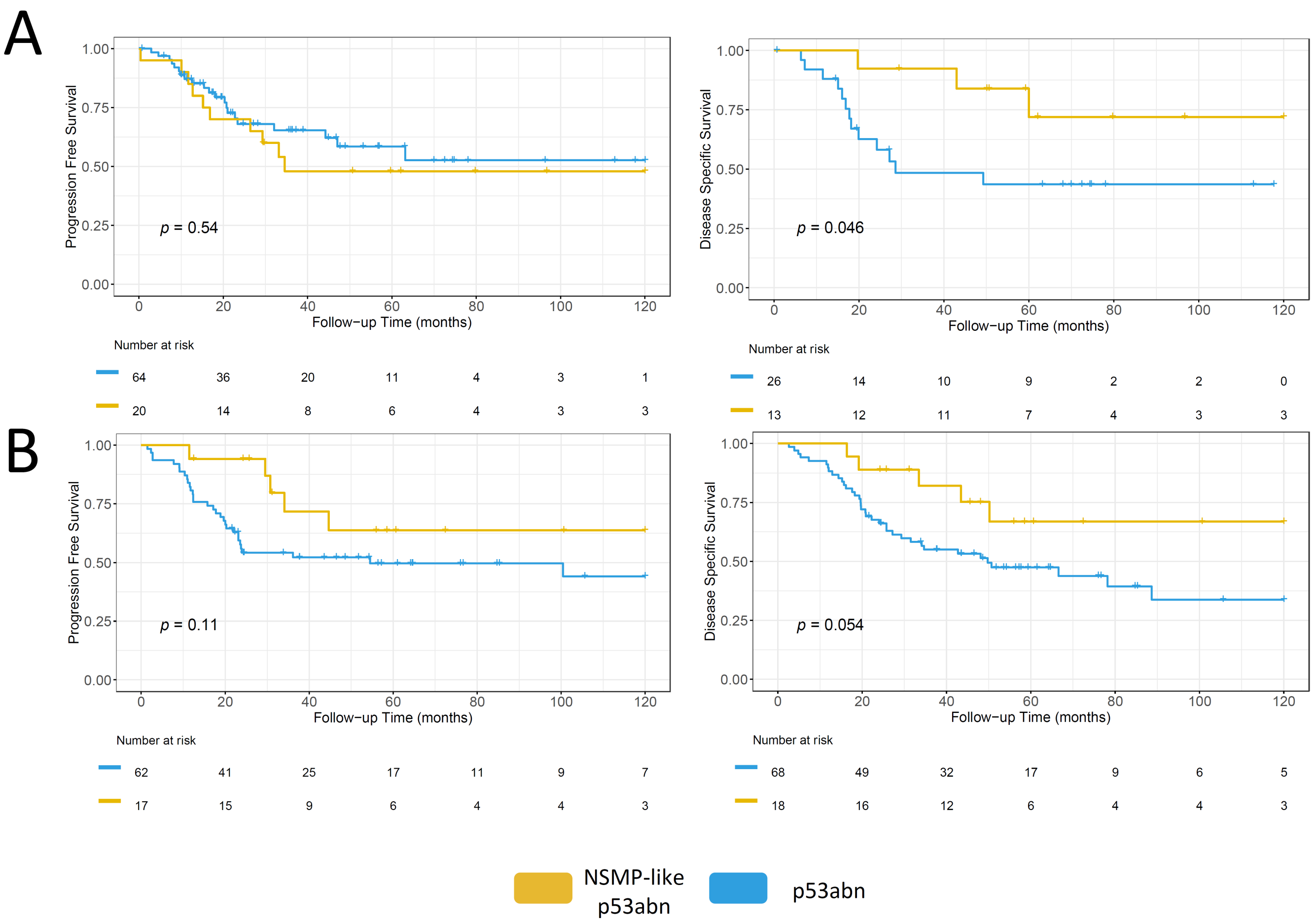

Supplementary Fig. 3: DSS and PFS KM curves associated with *NSMP-like p53abn* and p53abn cases in the (A) discovery set, and the (B) BC validation set. *NSMP-like p53abn* represents cases that are p53abn as assessed by IHC but classified as NSMP based on H&E slides by the AI model (The reported p-values compare the significance between p53abn-like NSMP and p53abn, computed using a two-sided log-rank statistical test).

A: Vanilla

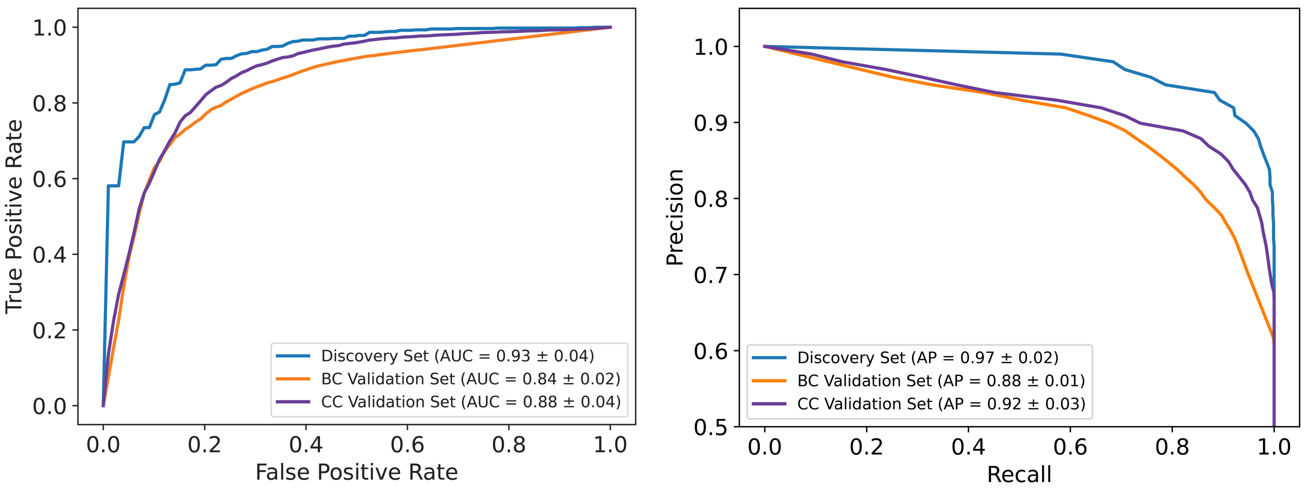

B: IDaRS

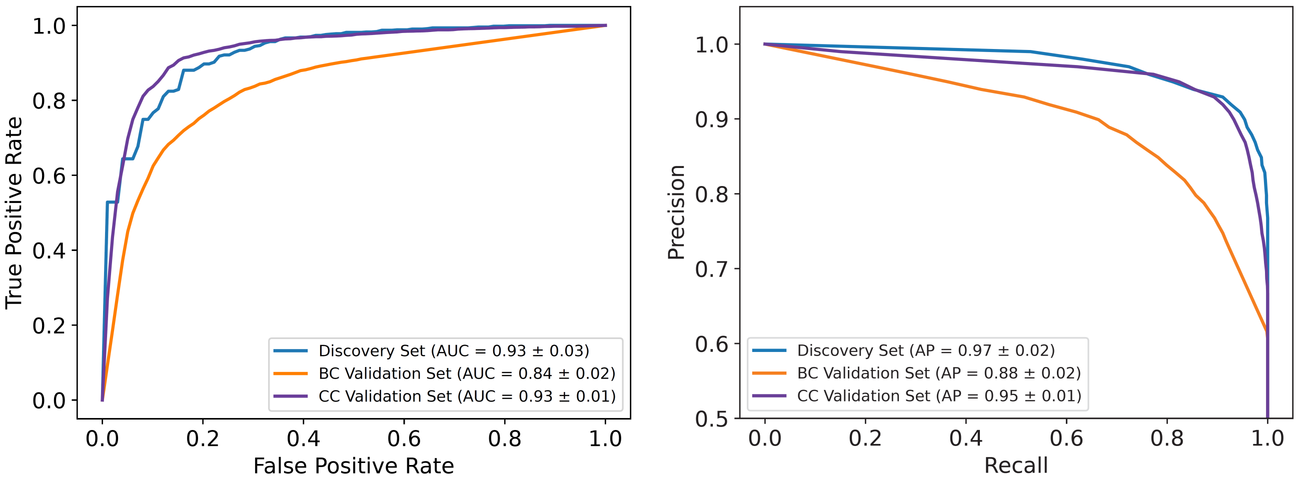

C: Histogram

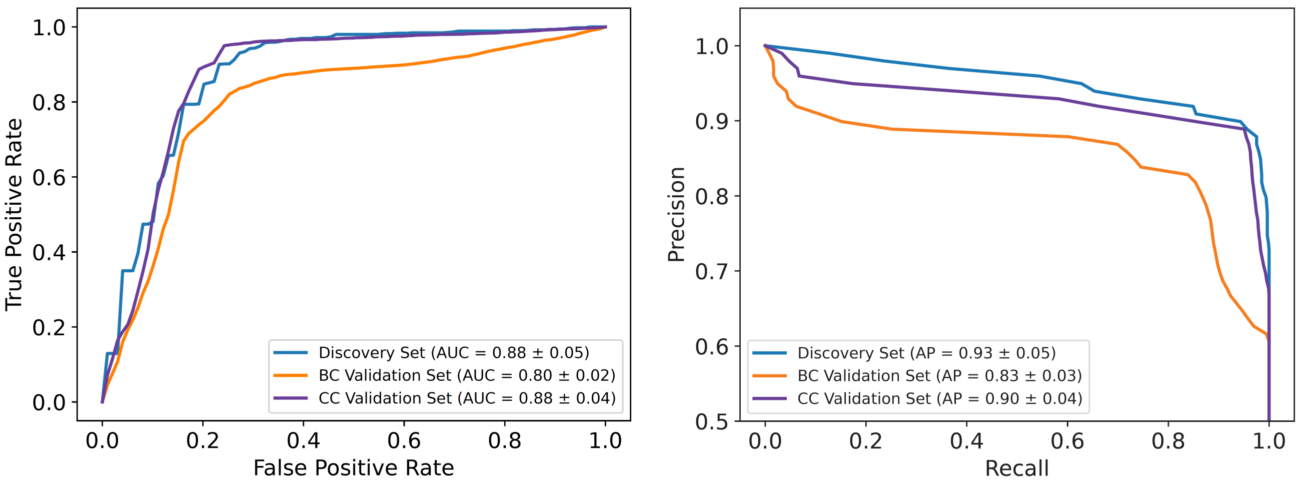

D: DeepMIL

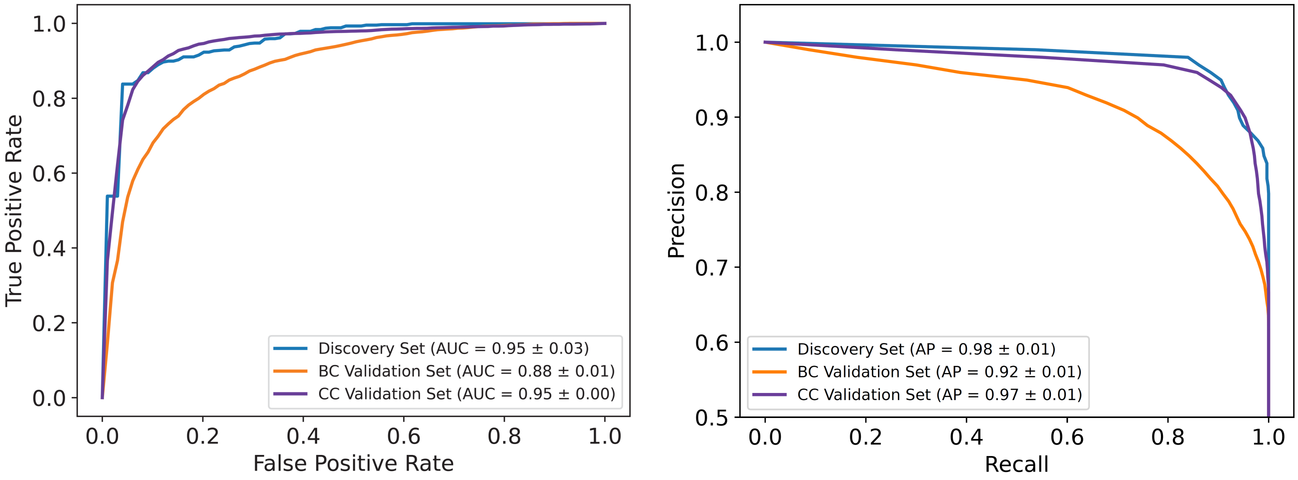

E: VLAD

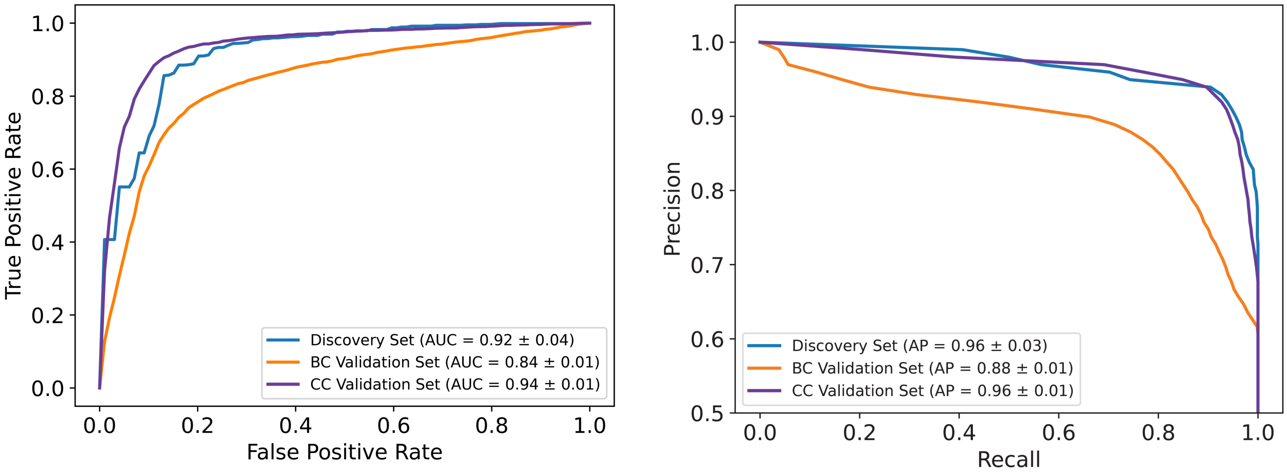

F: CLAM-MB

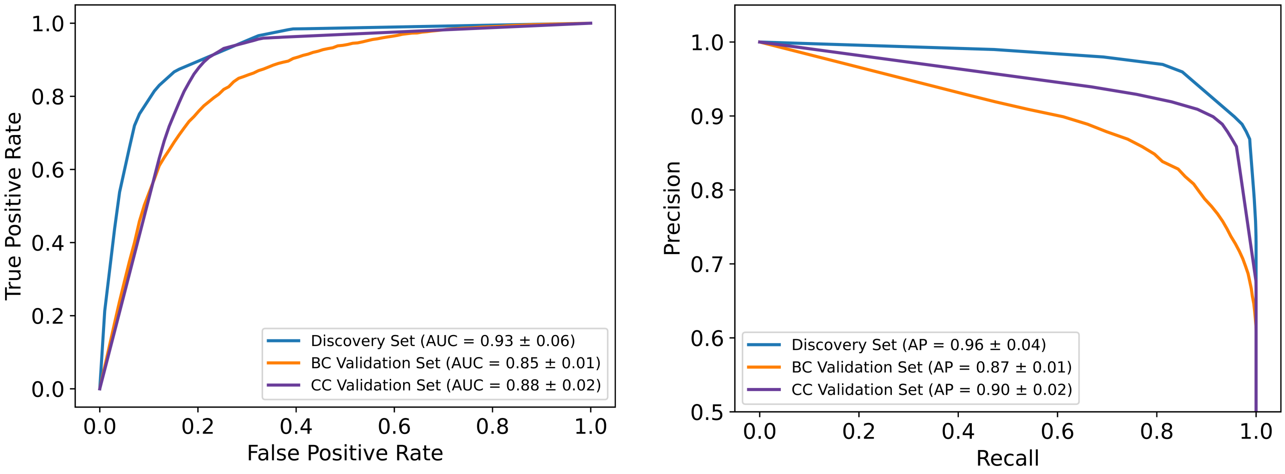

G: CLAM-SB

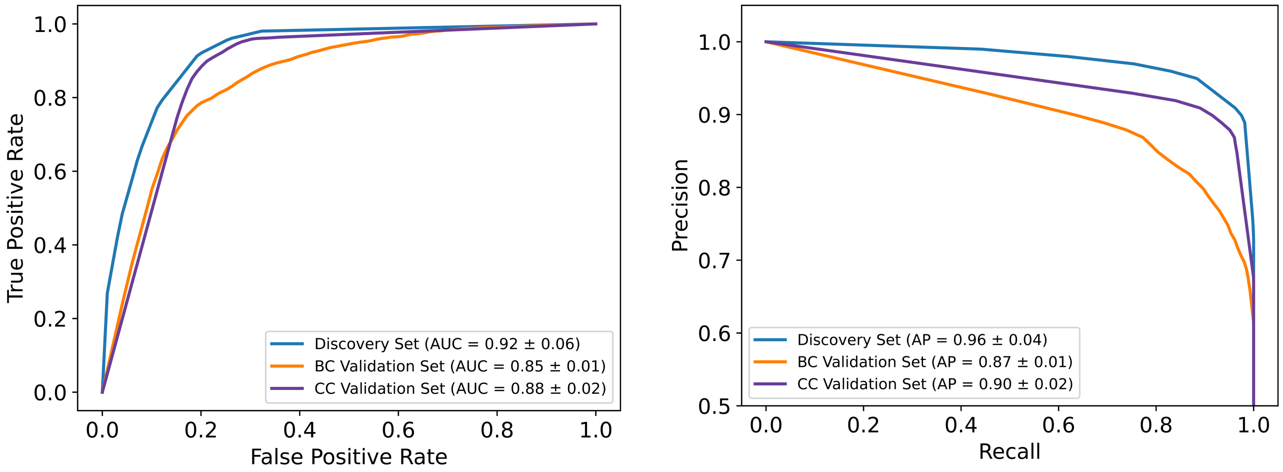

H: TransMIL

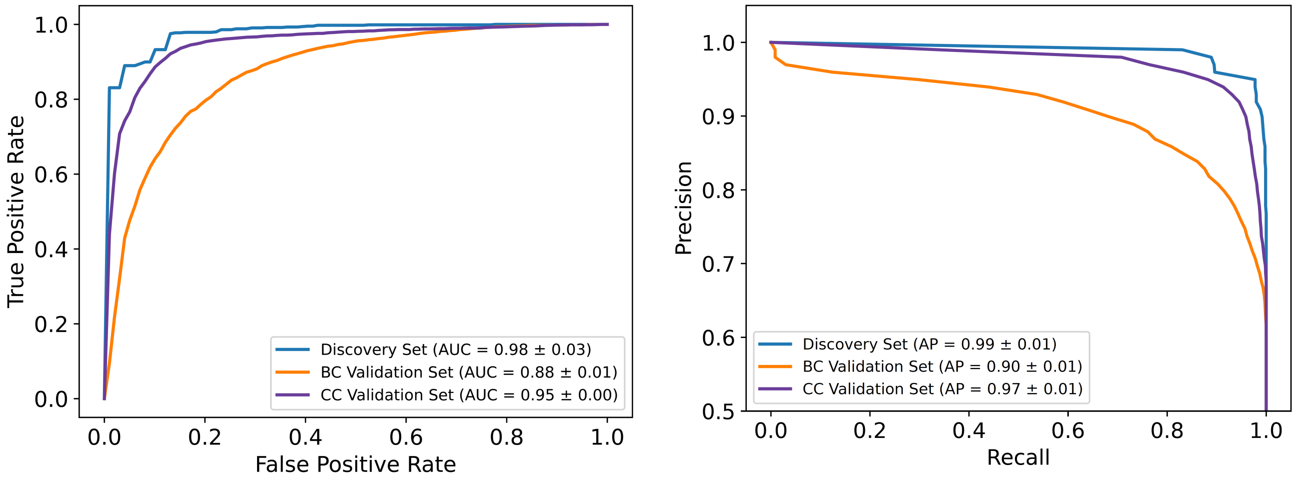

Supplementary Figure 4: Performance benchmarking of Vanilla (A), IDaRS (B), Histogram (C), DeepMIL (D), VLAD (E), CLAM-MB (F), CLAM-SB (G), and TransMIL(H) models for the discovery and validation sets.

A: Discovery cohort

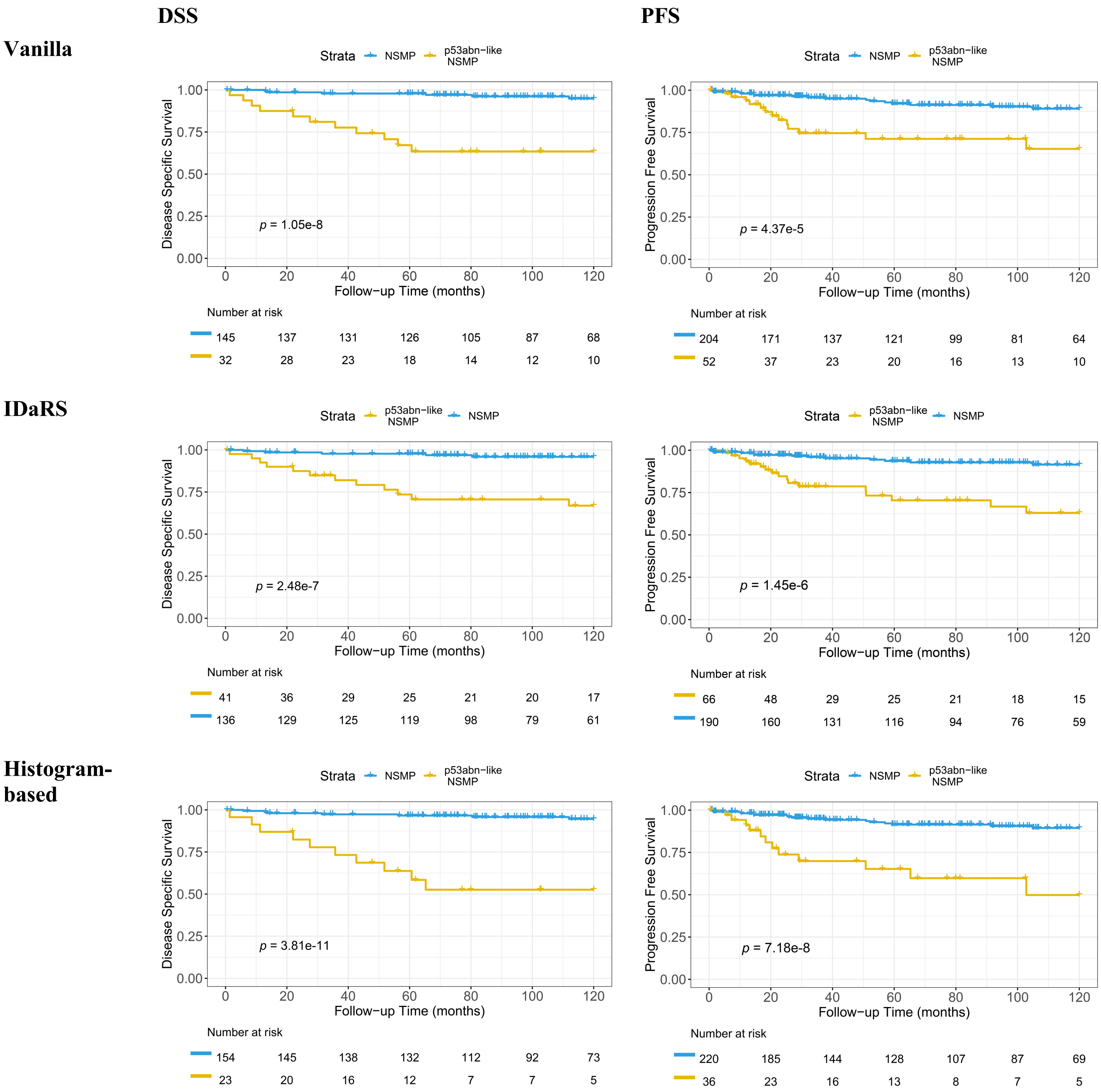

DeepMIL

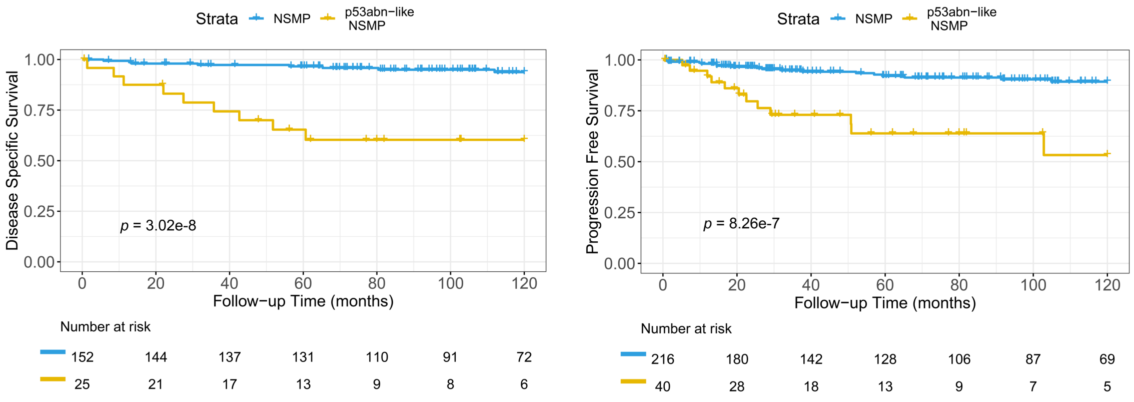

VLAD

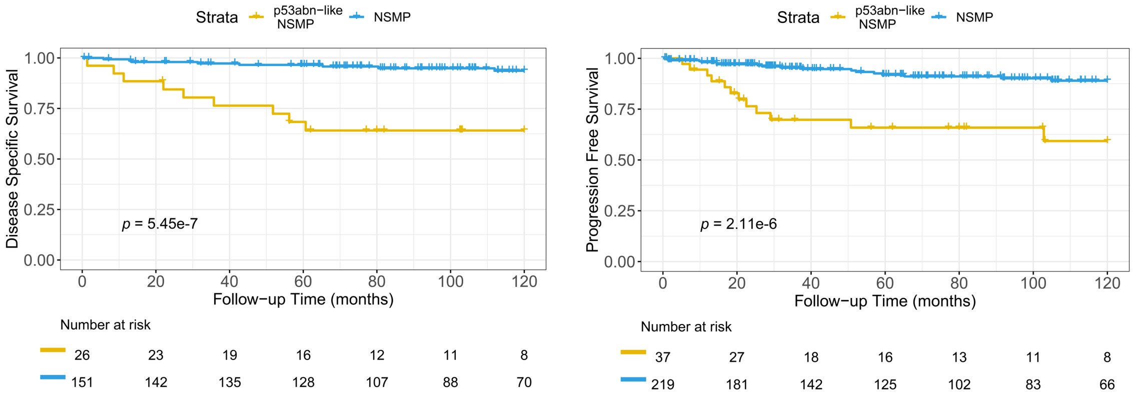

CLAM-MB

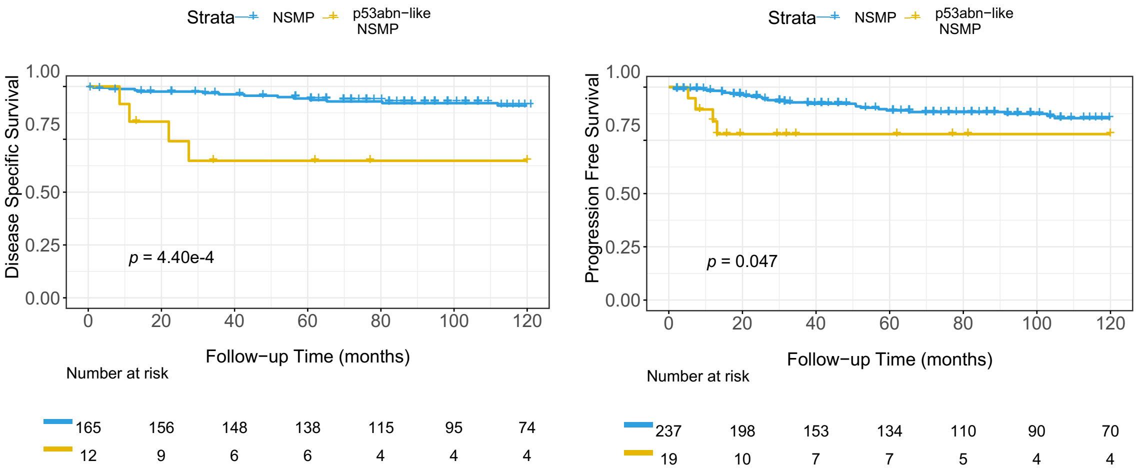

CLAM-SB

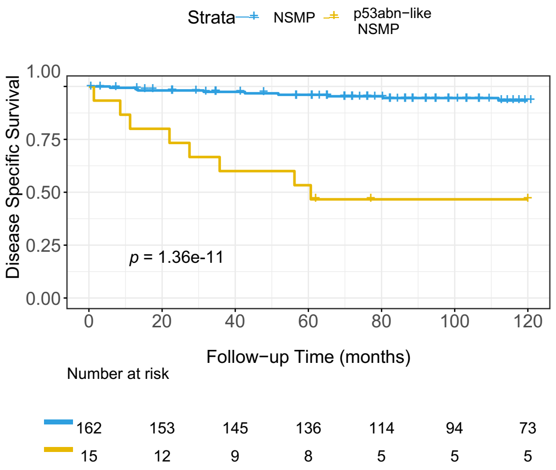

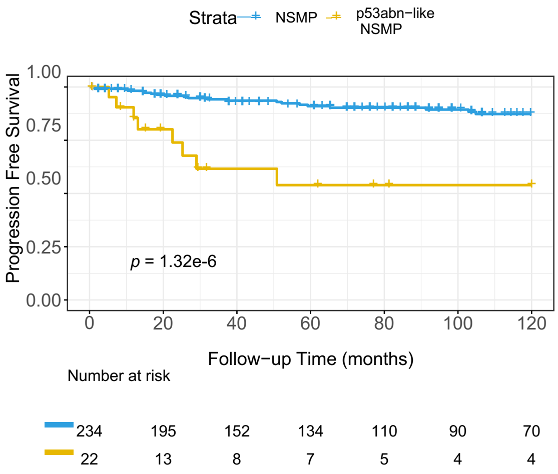

TransMIL

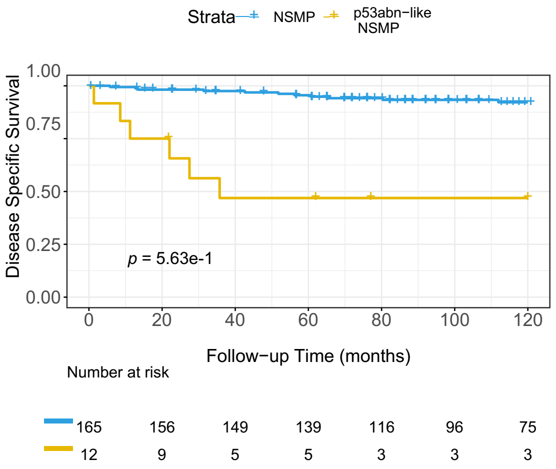

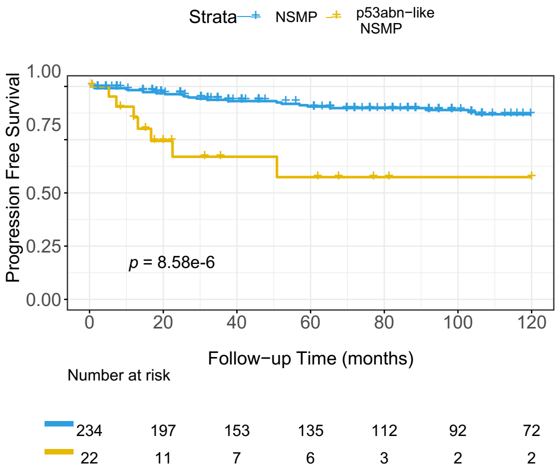

B: BC validation cohort

DSS

PFS

Vanilla

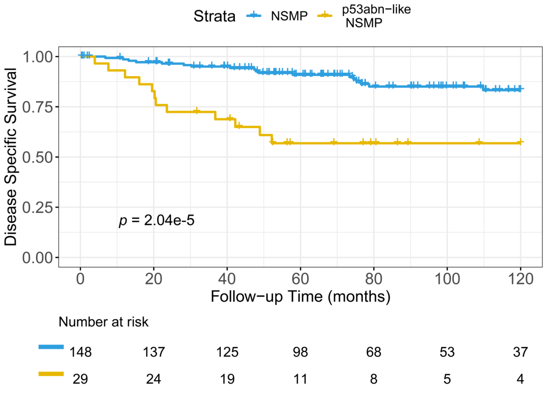

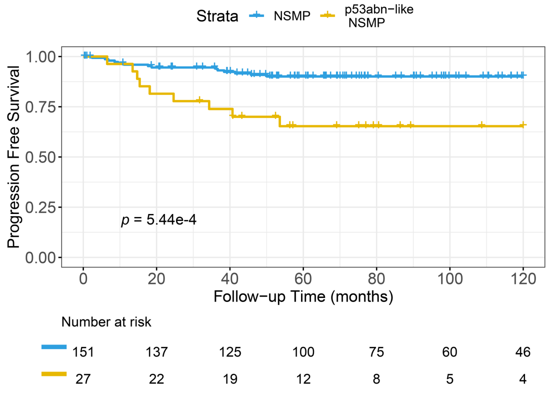

IDaRS

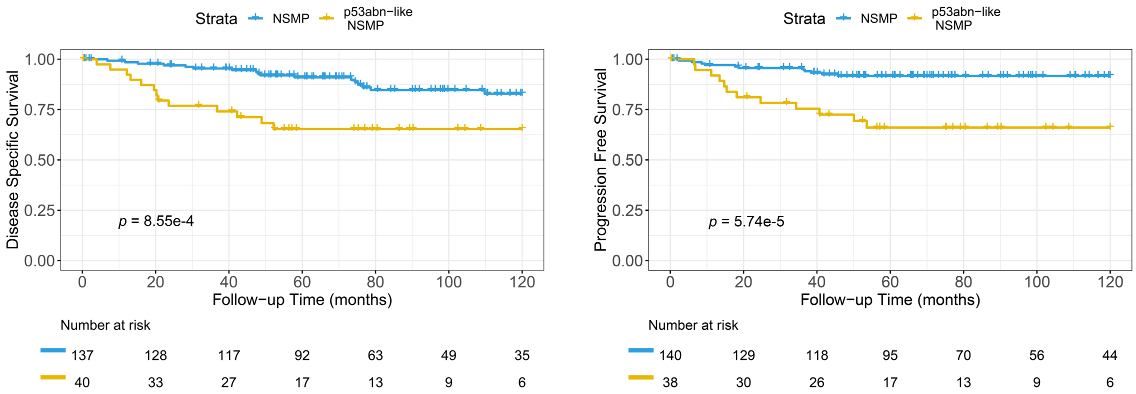

Histogram-based

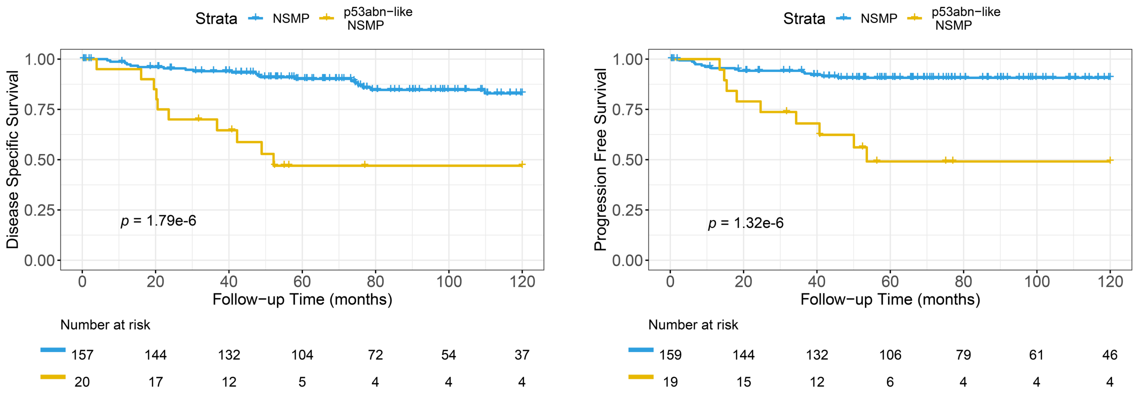

DeepMIL

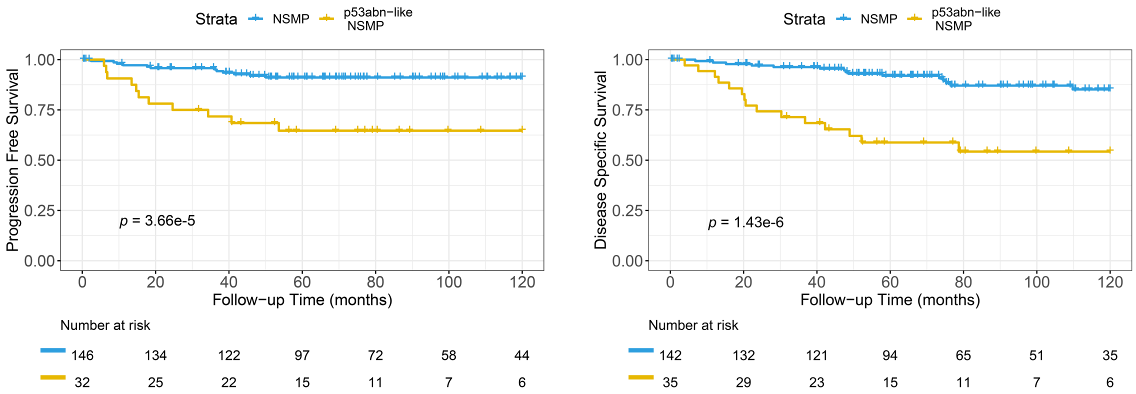

VLAD

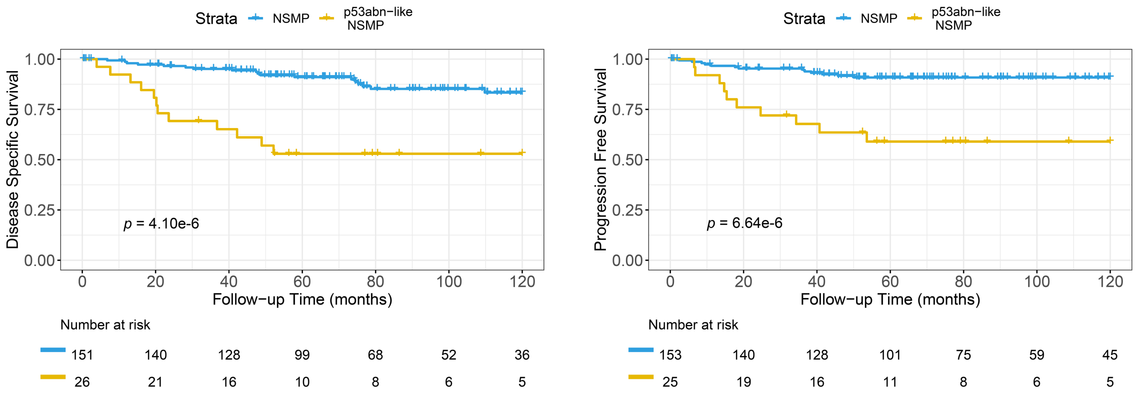

CLAM-MB

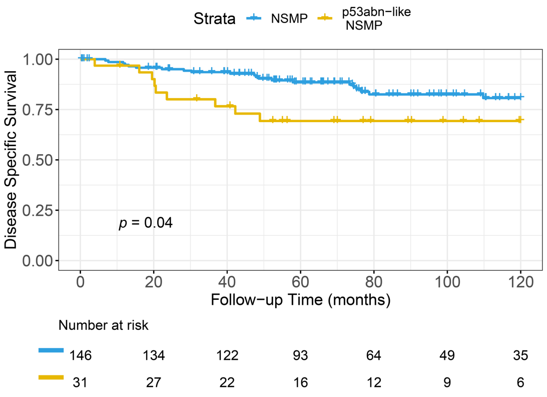

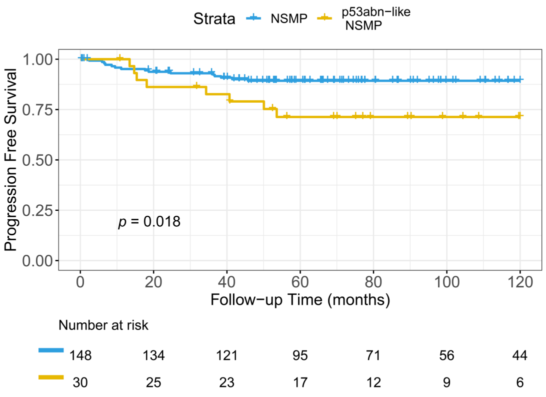

CLAM-SB

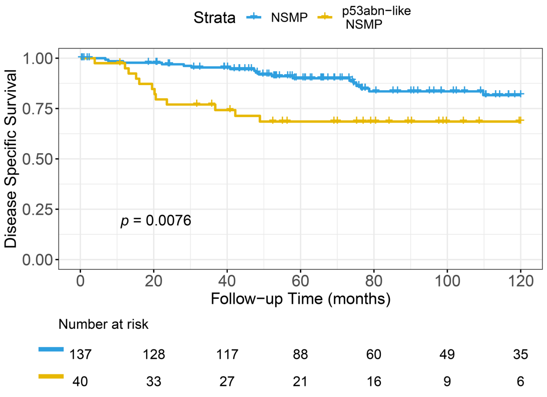

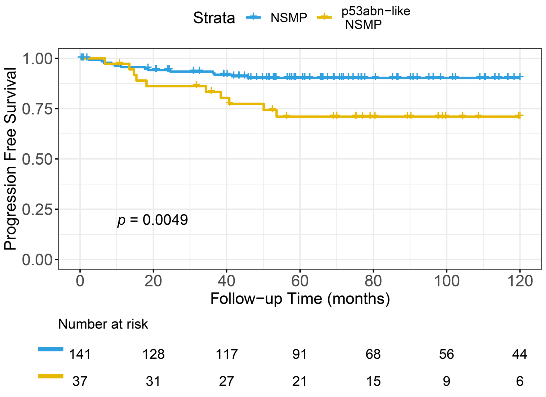

TransMIL

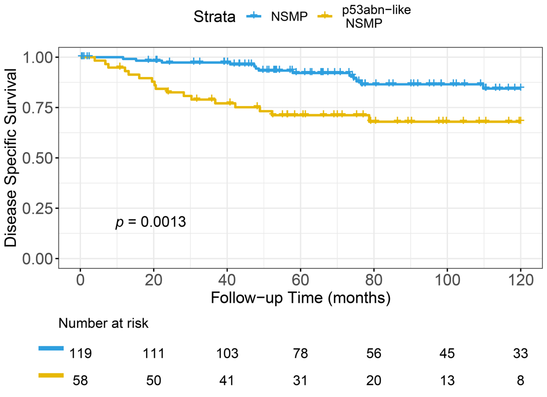

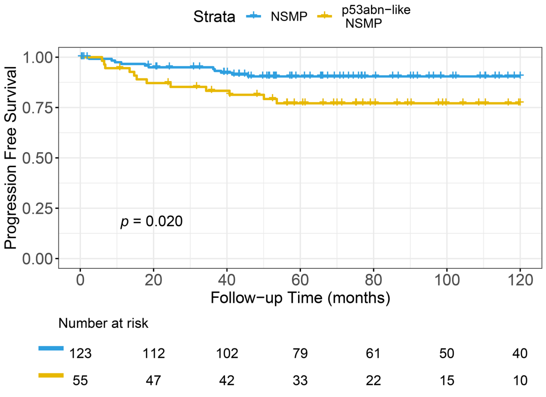

C: CC validation cohort

Vanilla

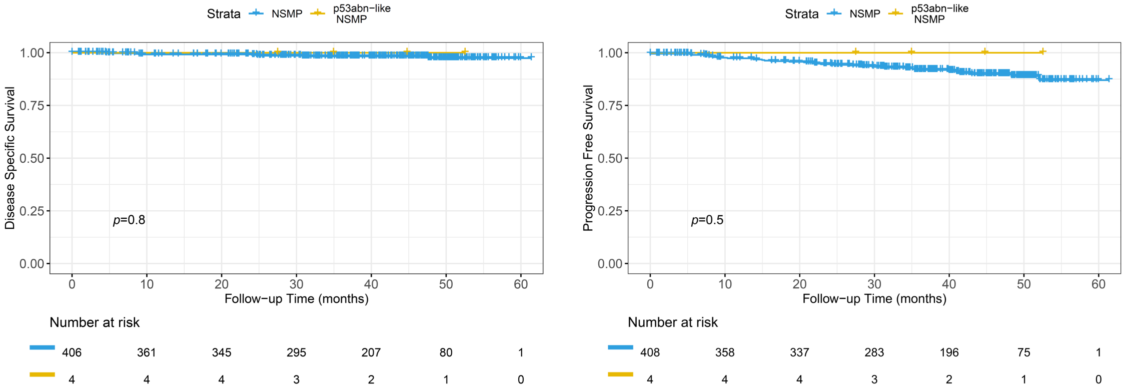

IDaRS

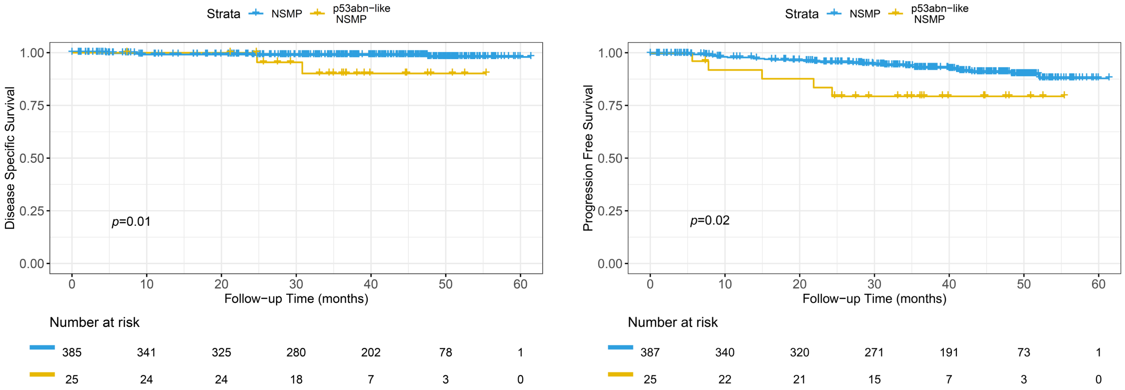

Histogram-based

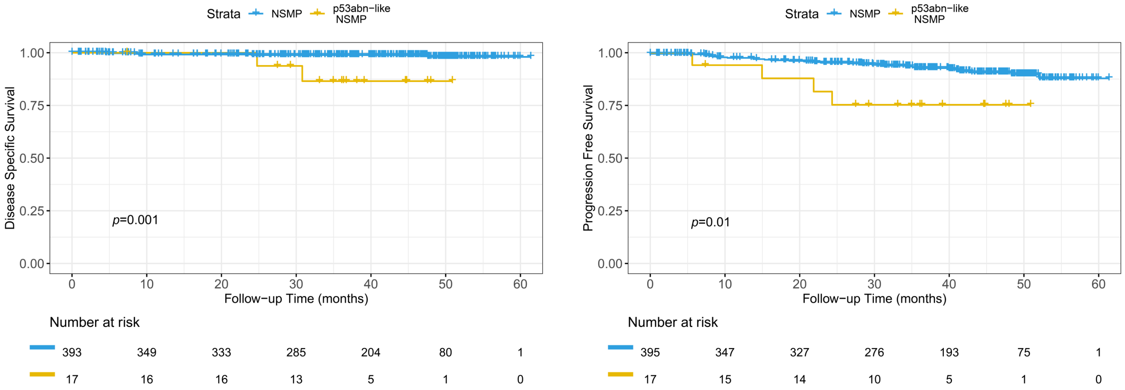

DeepMIL

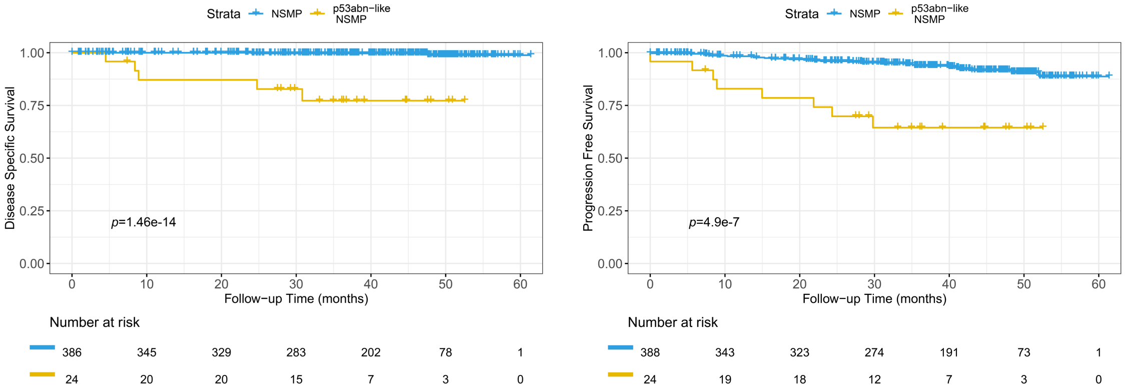

VLAD

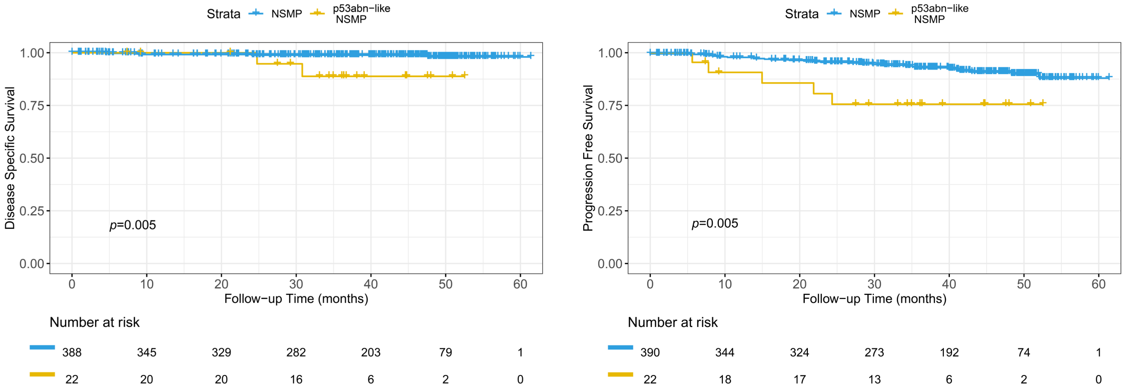

CLAM-MB

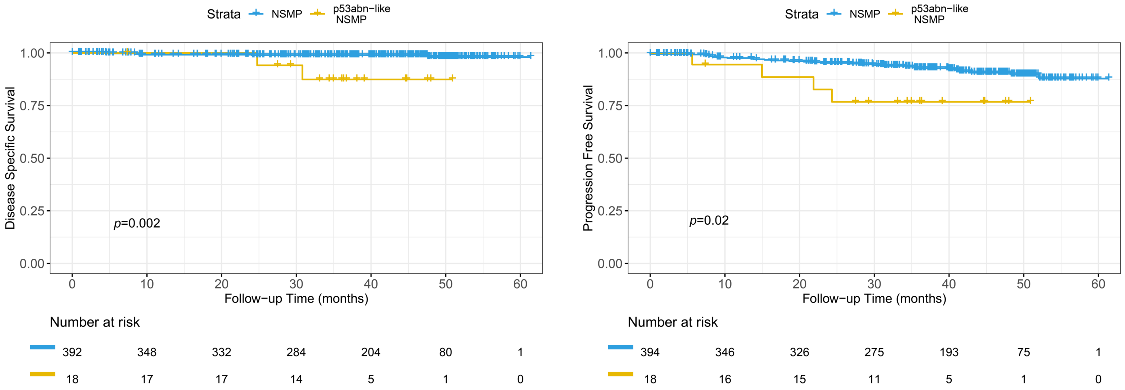

CLAM-SB

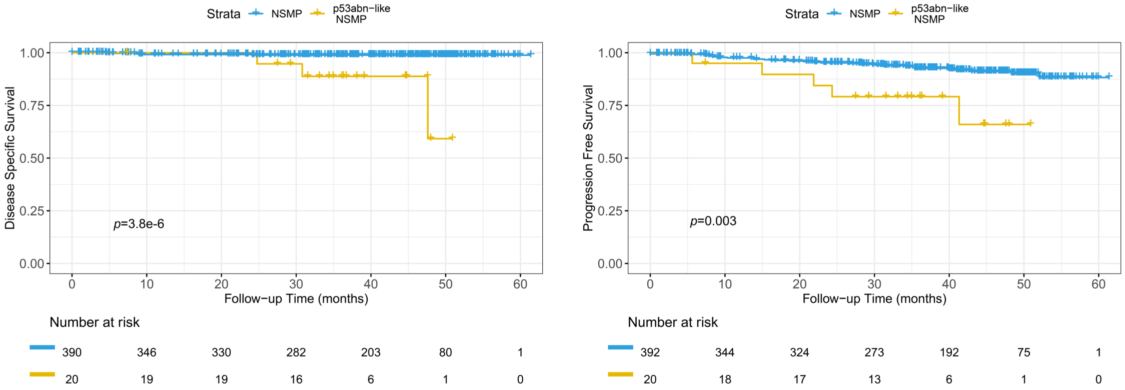

TransMIL

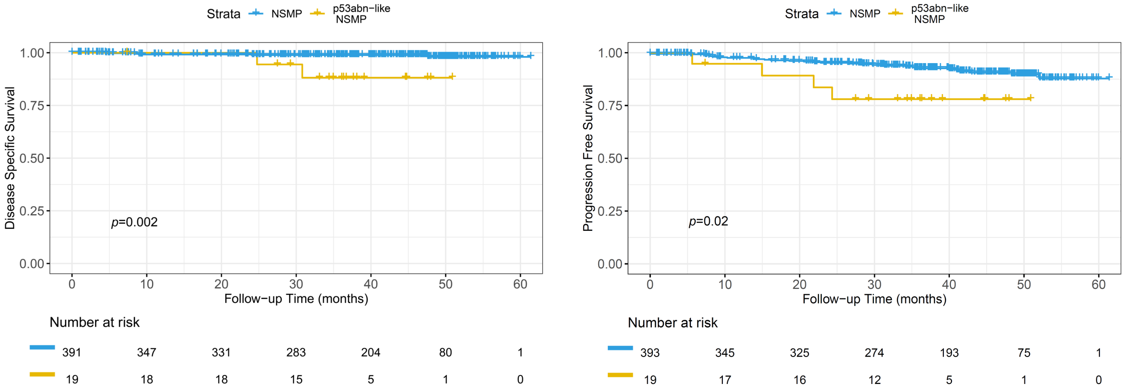

Supplementary Figure 5: Kaplan Meier curves along associated with the p53abn-like NSMP and NSMP groups for the (A) discovery, (B) BC validation, and CC validation cohorts using various deep learning frameworks. Note, DSS was not available for the TCGA part of the discovery cohort (The reported p-values compare the significance between p53abn-like NSMP and p53abn, computed using a two-sided log-rank statistical test).

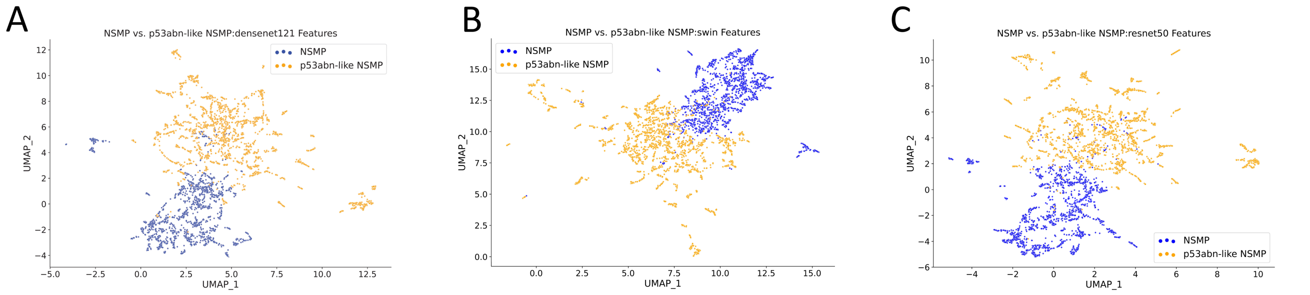

Supplementary Figure 6: Histopathological features from the slides in the BC validation cohort utilizing (A) Densnet121, (B) Swin, and (C) ResNet50 feature representations from the slides demonstrate that p53abn-like NSMP and the rest of the NSMP cases constitute two separate clusters.

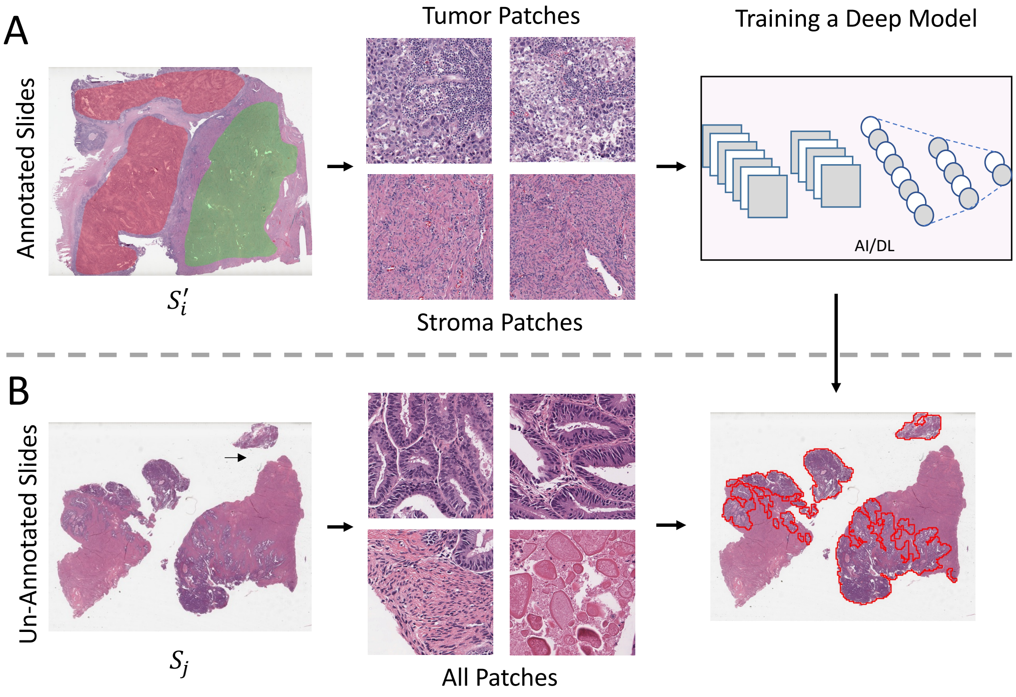

Supplementary Fig. 7: An overview of AI tumor-normal classifier and automatic annotation. (A) Extracting tumor and stroma patches from manually annotated slide and training a binary deep model. Red regions show annotated tumor regions, while green depicts stroma sections. (B) Extracting all patches from un-annotated slides and feeding them to the trained model to highlight only the tumor ones. The final tumor regions are shown by red contours.

Supplementary Table 1: Overview of cohorts.

Dataset	Unit	Subtype	
		**NSMP**	**p53abn**
TCGA (WSIs)	Patients	90	56
	Slides	94	61
	Manually Annotated Slides by Pathologists	14	13
	512×512 Pixel Patches at 20x	162,998	82,655
German (WSIs)	Patients	182	40
	Slides	355	76
	Manually Annotated Slides by Pathologists	0	0
	512×512 Pixel Patches at 20x	576,553	125,376
Vancouver (TMAs)	Patients	195	95
	Slides	670	422
	512×512 Pixel Patches at 20x with 65% Overlap	15,584	10,251
Cross Canada (WSIs)	Patients	416	198
	Slides	432	208
	Manually Annotated Slides by Pathologists	0	0
	512×512 Pixel Patches at 20x	696,340	414,019

Supplementary Table 2: Outcome information of the discovery and validation cohorts.

Source			NSMP	*p53abn-like NSMP*	p53abn
Discovery Cohort	Disease Specific (German)	Total	155 (69.82%)	27 (12.16%)	40 (18.02%)
		Censored	143 (92.26%)	17 (62.96%)	23 (57.50%)
		Event	8 (5.16%)	9 (33.33%)	16 (40.00%)
		Unk	4 (2.58%)	1 (3.70%)	1 (2.50%)
	Progression Free (German and TCGA)	Total	221 (60.88%)	47 (12.95%)	95 (26.17%)
		Censored	197 (89.14%)	30 (63.82%)	53 (55.79%)
		Event	16 (7.24%)	13 (27.66%)	31 (32.63%)
		Unk	8 (3.62%)	4 (8.51%)	11 (11.58%)
BC Validation Cohort	Disease Specific	Total	155 (53·82%)	38 (13·19%)	95 (32·99%)
		Censored	125 (80.65%)	22 (57.89%)	44 (46.32%)
		Event	16 (10.32%)	14 (36.84%)	42 (44.21%)
		Unk	14 (9.03%)	2 (5.26%)	9 (9.47%)
	Progression Free	Total	155 (53·82%)	38 (13·19%)	95 (32·99%)
		Censored	133 (85.81%)	22 (57.89%)	43 (45.26%)
		Event	12 (7.74%)	11 (28.95%)	36 (37.89%)
		Unk	10 (6.45%)	5 (13.16%)	16 (16.82%)
CC Validation Cohort	Disease Specific	Total	390 (63.52%)	26 (4.23%)	198 (32.25%)
		Censored	382 (97.95%)	21 (80.87%)	165 (80.05%)
		Event	2 (0.51%)	5 (19.23%)	29 (14.95%)
		Unk	6 (1.54%)	0	4 (2.06%)
	Progression Free	Total	390 (63.52%)	26 (4.23%)	198 (32.25%)
		Censored	360 (92.31%)	18 (69.23%)	135 (68.18%)
		Event	26 (6.67%)	8 (30.77%)	63 (31.82%)
		Ukn	4 (1.03%)	0	0

Supplementary Table 3: Treatment information of the discovery and validation cohorts.

	Treatment	Total	NSMP	p53abn
Discovery Cohort (German)	Total	217	178	39
	None	76 (32.02)	68 (37.99%)	8 (20.51%)
	RT only	8 (3.69%)	6 (3.35%)	2 (5.13%)
	brachy only	66 (30.41%)	63 (35.20%)	3 (7.69%)
	brachy+RT	4 (1.84%)	3 (1.68%)	1 (2.56%)
	chemo only	23 (10.60%)	9 (5.03%)	14 (35.90%)
	chemo+RT	5 (2.30%)	3 (1.68%)	2 (5.13%)
	chemo+brach+RT	1 (0.46%)	1 (0.56%)	0
	chemo+brachy	3 (1.38%)	1 (0.56%)	2 (5.13%)
	N / A	31 (14.29%)	24 (13.41%)	7 (17.95%)
BC Validation Cohort	Total	288	193	95
	None	158 (54.86%)	128 (66.32%)	30 (31.58%)
	Rt only	47 (16.32%)	31 (16.06%)	16 (16.84%)
	Brachy only	5 (1.74%)	4 (2.07%)	1 (1.05%)
	Chemo only	27 (9.38%)	6 (3.11%)	21 (22.11%)
	Chemo + RT	43 (14.93%)	21 (10.88%)	22 (23.16%)
	N / A	8 (2.78%)	3 (1.55%)	5 (5.26%)
CC Validation Cohort	Total	614	416	198
	None	342 (55.7%)	284 (68.27%)	58 (29.29%)
	RT only	28 (4.56%)	21 (5.05%)	7 (3.54%)
	brachy only	66 (10.75%)	53 (12.74%)	13 (6.57%)
	chemo+brachy+RT	26 (4.23%)	10 (2.4%)	16 (8.08%)
	chemo+brachy	33 (5.37%)	5 (1.2%)	28 (14.14%)
	chemo+RT	56 (9.12%)	20 (4.81%	36 (18.18%)
	brachy+RT	13 (2.12%)	9 (2.16%)	4 (2.02%)
	chemo only	46 (7.49%)	11 (2.64%)	35 (17.68%)
	chemo+brachy+RT+boost	3 (0.49%)	2 (0.48%)	1 (0.51%)
	horm only	1 (0.16%)	1 (0.24%)	0

Supplementary Table 4: Distribution of samples across different centres in the CC validation cohort.

Center	Total	NSMP	P53abn
Total	614	416	198
LGH	5 (0.81%)	4 (0.96%)	1 (0.51%)
HAM	65 (10.59%)	25 (6.01%)	40 (20.2%)
ARH	8 (1.3%)	8 (1.92%)	0 (0.0%)
CHM	19 (3.09%)	14 (3.37%)	5 (2.53%)
CMX	1 (0.16%)	1 (0.24%)	0 (0.0%)
LAV	104 (16.94%)	75 (18.03%)	29 (14.65%)
RGH	3 (0.49%)	3 (0.72%)	0 (0.0%)
PEN	2 (0.33%)	2 (0.48%)	0 (0.0%)
NAN	6 (0.98%)	5 (1.2%)	1 (0.51%)
RJH	27 (4.4%)	21 (5.05%)	6 (3.03%)
SSK	18 (2.93%)	12 (2.88%)	6 (3.03%)
KAM	4 (0.65%)	3 (0.72%)	1 (0.51%)
BBY	2 (0.33%)	2 (0.48%)	0 (0.0%)
VER	1 (0.16%)	1 (0.24%)	0 (0.0%)
JGH	39 (6.35%)	26 (6.25%)	13 (6.57%)
KEL	4 (0.65%)	4 (0.96%)	0 (0.0%)
WPG	19 (3.09%)	15 (3.61%)	4 (2.02%)
CRN	1 (0.16%)	1 (0.24%)	0 (0.0%)
UNH	5 (0.81%)	5 (1.2%)	0 (0.0%)
RCH	12 (1.95%)	12 (2.88%)	0 (0.0%)
NSH	56 (9.12%)	47 (11.3%)	9 (4.55%)
SUN	115 (18.73%)	55 (13.22%)	60 (30.3%)
VGH	81 (13.19%)	58 (13.94%)	23 (11.62%)
SPH	8 (1.3%)	8 (1.92%)	0 (0.0%)
TRL	2 (0.33%)	2 (0.48%)	0 (0.0%)
SMH	7 (1.14%)	7 (1.68%)	0 (0.0%)

Supplementary Table 5: Accuracy and other performance measures of the binary tumor-stroma classifier.

Non-tumor Accuracy	Tumor Accuracy	Kappa	F1 Score	AUC	Balanced Accuracy
99·89%	99·61%	0·9952	0·9976	0·9994	99·75%

Supplementary Table 6: Performance metrics of the deep learning model for p53abn vs. NSMP classifier (based on VarMIL method). The results are based on mean ± std of 10 cross-validation splits.

Source	p53abn Accuracy (%)	NSMP Accuracy (%)	Kappa	F1 score	AUC	Balanced Accuracy (%)
Discovery Set	87·17 ± 10·86	91·59 ± 3·58	0·75 ± 0·09	0·87 ± 0·05	0·95 ± 0·03	89·38 ± 5·49
BC Validation Set	81·99 ± 4·56	77·61 ± 5·62	0·58 ± 0·03	0·79 ± 0·02	0·88 ± 0·01	79·80 ± 1·34
CC Validation Set	84.76 ± 3.48	92.24 ± 2.1	0.77 ± 0.02	0.88 ± 0.01	0.95 ± 0.0	88.5 ± 1.08

Supplementary Table 7: Detailed accuracy and other performance measures of the VarMIL network.

Source	Split	Metrics					
		p53abn Accuracy (%)	NSMP Accuracy (%)	Kappa	F1 score	AUC	Average Score (%)
Discovery Set	1	70·97	90·48	0·6208	0·8104	0·9078	80·72
	2	64·52	89·41	0·5506	0·7752	0·9120	76·96
	3	96·00	93·33	0·8332	0·9164	0·9867	94·67
	4	92·59	91·67	0·7928	0·8961	0·9749	92·13
	5	96·30	85·71	0·7205	0·8586	0·9616	91·01
	6	96·30	89·41	0·7784	0·8885	0·9760	92·85
	7	96·15	87·76	0·7262	0·8617	0·9380	91·95
	8	88·46	94·90	0·8107	0·9053	0·9686	91·68
	9	80·77	97·98	0·8225	0·9112	0·9526	89·37
	10	89·66	95·29	0·8400	0·9200	0·9542	92·47
BC Validation Set	1	85·78	75·82	0·5897	0·7930	0·8836	80·80
	2	81·28	81·19	0·6121	0·8057	0·8822	81·24
	3	81·52	82·24	0·6263	0·8129	0·8972	81·88
	4	89·34	70·60	0·5619	0·7767	0·8869	79·97
	5	79·86	81·49	0·6032	0·8013	0·8892	80·68
	6	76·07	81·79	0·5733	0·7866	0·8766	78·93
	7	90·05	64·03	0·4974	0·7403	0·8679	77·04
	8	78·67	79·70	0·5722	0·7857	0·8755	79·19
	9	77·96	79·70	0·5660	0·7826	0·8731	78·83
	10	79·38	79·55	0·5767	0·7879	0·8742	79·47
CC Validation Set	1	81.07	94.17	0.7659	0.8829	0.9428	87.62
	2	84.47	93.01	0.7767	0.8883	0.9491	88.74
	3	85.92	93.24	0.7916	0.8958	0.9545	89.58
	4	89.81	91.38	0.7957	0.8978	0.9507	90.59
	5	78.64	94.64	0.7527	0.8762	0.9471	86.64
	6	86.41	91.14	0.7658	0.8829	0.9439	88.77
	7	89.32	88.34	0.7502	0.8748	0.9430	88.83
	8	85.92	91.84	0.7718	0.8859	0.9418	88.88
	9	85.44	89.51	0.7356	0.8677	0.9394	87.47
	10	80.58	95.10	0.7755	0.8876	0.9526	87.84

Supplementary Table 8: Statistics of NSMP and *p53abn-like NSMP* patients based on p53abn vs. NSMP classifiers.

Source	Number of patients	NSMP	*p53abn-like NSMP*
Discovery Set	272	224 (82·35%)	48 (17·65%)
BC Validation Set	195	156 (80%)	39 (20%)
CC Validation Set	416	390 (93.75%)	26 (6.25%)

Supplementary Table 9: Performance benchmarking of Vanilla (A), IDaRS (B), Histogram (C), DeepMIL (D), VLAD (E), CLAM-MB (F), CLAM-SB (G), and TransMIL (H) models for the discovery and validation sets.

A: Vanilla

Source	P53abn Accuracy (%)	NSMP Accuracy (%)	Kappa	F1 score	AUC	Balanced Accuracy (%)
Discovery Set	74.80 ± 11.91	92.28 ± 5.51	0.68 ± 0.10	0.84 ± 0.05	0.93 ± 0.04	83.54 ± 5.69
BC Validation Set	79.46 ± 7.51	75.22 ± 11.43	0.53 ± 0.07	0.76 ± 0.04	0.84 ± 0.02	77.34 ± 2.85
CC Validation Set	57.96 ± 30.74	74.69 ± 34.19	0.34 ± 0.18	0.62 ± 0.15	0.88 ± 0.04	66.32 ± 8.6

B: IDaRS

Source	p53abn Accuracy (%)	NSMP Accuracy (%)	Kappa	F1 score	AUC	Balanced Accuracy (%)
Discovery Set	81.63 ± 9.78	88.61 ± 5.22	0.66 ± 0.07	0.83 ± 0.04	0.93 ± 0.03	85.12 ± 3.98
BC Validation Set	83.41 ± 5.21	71.19 ± 10.90	& 0.52 ± 0.08	0.75 ± 0.05	0.84 ± 0.02	77.30 ± 3.34
CC Validation Set	86.07± 3.56	86.20± 10.41	0.7024± 0.1	0.85± 0.06	0.93± 0.01	86.13± 3.56

C: Histogram

Source	p53abn Accuracy (%)	NSMP Accuracy (%)	Kappa	F1 score	AUC	Balanced Accuracy (%)
Discovery Set	72.38 ± 10.26	94.83 ± 2.64	0.70 ± 0.10	0.85 ± 0.05	0.88 ± 0.05	83.60 ± 5.34
BC Validation Set	75.88 ± 5.58	81.04 ± 4.95	0.56 ± 0.04	0.78 ± 0.02	0.80 ± 0.02	78.46 ± 1.77
CC Validation Set	80.68 ± 5.65	93.68 ± 1.72	0.76 ± 0.03	0.88 ± 0.02	0.92 ± 0.01	87.18 ± 2.29

D: DeepMIL

Source	p53abn Accuracy (%)	NSMP Accuracy (%)	Kappa	F1 score	AUC	Balanced Accuracy (%)
Discovery Set	85.72 ± 11.55	91.45 ± 4.31	0.74 ± 0.12	0.87 ± 0.06	0.95 ± 0.03	88.58 ± 6.60
BC Validation Set	82.84 ± 4.15	77.60 ± 5.65	0.59 ± 0.03	0.79 ± 0.02	0.88 ± 0.01	80.22 ± 1.32
CC Validation Set	83.69± 1.77	93.26± 0.83	0.7741± 0.01	0.89± 0	0.95± 0	88.48± 0.61

E: VLAD

Source	p53abn Accuracy (%)	NSMP Accuracy (%)	Kappa	F1 score	AUC	Balanced Accuracy (%)
Discovery Set	79.11 ± 6.67	93.43 ± 2.45	0.73 ± 0.08	0.86 ± 0.04	0.92± 0.04	86.27 ± 3.87
BC Validation Set	77.48 ± 4.56	80.36 ± 3.50	0.57 ± 0.03	0.78 ± 0.01	0.84 ± 0.01	78.92 ± 1.53
CC Validation Set	83.18 ± 2.09	92.27 ± 1.14	0.76 ± 0.02	0.88 ± 0.01	0.93 ± 0.01	87.72 ± 1.05

F: CLAM-MB

Source	p53abn Accuracy (%)	NSMP Accuracy (%)	Kappa	F1 score	AUC	Balanced Accuracy (%)
Discovery Set	86.53 ± 11.8	97.72 ± 1.42	0.86 ± 0.1	0.93 ± 0.05	0.93 ± 0.06	92.12 ± 6.06
BC Validation Set	77.15 ± 7.05	79.63 ± 7.93	0.56 ± 0.04	0.78 ± 0.02	0.85 ± 0.01	78.39 ± 1.33
CC Validation Set	78.59 ± 5.61	94.22 ± 2.29	0.75 ± 0.03	0.87 ± 0.01	0.88 ± 0.02	86.41 ± 1.94

G: CLAM-SB

Source	p53abn Accuracy (%)	NSMP Accuracy (%)	Kappa	F1 score	AUC	Balanced Accuracy (%)
Discovery Set	86.97 ± 10.36	97.51 ± 2.78	0.86 ± 0.1	0.93 ± 0.05	0.92 ± 0.06	92.24 ± 5.56
BC Validation Set	78.13 ± 6.8	79.65 ± 6.49	0.57 ± 0.03	0.78 ± 0.01	0.85 ± 0.01	78.89 ± 1.12
CC Validation Set	78.25 ± 5.1	94.97 ± 1.32	0.75 ± 0.03	0.88 ± 0.01	0.88 ± 0.02	86.61 ± 1.99

H: TransMIL

Source	p53abn Accuracy (%)	NSMP Accuracy (%)	Kappa	F1 score	AUC	Balanced Accuracy (%)
Discovery Set	92.45 ± 8.39	97.46 ± 2.8	0.90 ± 0.	0.95 ± 0.05	0.98 ± 0.03	94.95 ± 5.29
BC Validation Set	82.44 ± 13.93	70.39 ± 13.97	0.5 ± 0.09	0.74 ± 0.05	0.88 ± 0.01	76.41 ± 4.32
CC Validation Set	81.46 ± 4.59	94.48 ± 1.64	0.77 ± 0.02	0.89 ± 0.01	0.95 ± 0	87.97 ± 1.54

Supplementary Table 10: Statistics of NSMP and p53abn-like NSMP patients based on p53abn vs. NSMP Vanilla (A), IDaRS (B), Histogram (C), DeepMIL (D), VLAD (E), CLAM-MB (F), CLAM-SB (G), and TransMIL (H) models for the discovery and validation sets.

A: Vanilla

Source	Number of patients	NSMP	p53abn-like NSMP
Discovery Set	272	213 (78.31%)	59 (21.69%)
BC Validation Set	195	163 (83.59%)	32 (16.41%)
CC Validation Set	416	412 (99.04%)	4 (0.96%)

B: IDaRS

Source	Number of patients	NSMP	p53abn-like NSMP
Discovery Set	272	200 (73.53%)	72 (26.47%)
BC Validation Set	195	152 (77.95%)	43 (22.05%)
CC Validation Set	416	391 (93.99%)	25 (6.01%)

C: Histogram

Source	Number of patients	NSMP	p53abn-like NSMP
Discovery Set	272	232 (85.29%)	40 (14.71%)
BC Validation Set	195	172 (88.21%)	23 (11.79%)
CC Validation Set	416	399 (95.91%)	17 (4.09%)

D: DeepMIL

Source	Number of patients	NSMP	p53abn-like NSMP
Discovery Set	272	228 (83.82%)	44 (16.18%)
BC Validation Set	195	157 (80.51%)	38 (19.49%)
CC Validation Set	416	392 (94.23%)	24 (5.77%)

E: VLAD

Source	Number of patients	NSMP	p53abn-like NSMP
Discovery Set	272	232 (85.29%)	40 (14.71%)
BC Validation Set	195	166 (85.13%)	29 (14.87%)
CC Validation Set	416	394 (94.71%)	22 (5.29%)

F: CLAM-MB

Source	Number of patients	NSMP	p53abn-like NSMP
Discovery Set	272	251 (92.28%)	21 (7.72%)

BC Validation Set	195	161 (82.56%)	34 (17.44%)
CC Validation Set	416	398 (95.67%)	18 (4.33%)

F: CLAM-SB

Source	Number of patients	NSMP	p53abn-like NSMP
Discovery Set	272	247 (90.81%)	25 (9.19%)
BC Validation Set	195	152 (77.95%)	43 (22.05%)
CC Validation Set	416	396 (95.19%)	20 (4.81%)

F: TransMIL

Source	Number of patients	NSMP	p53abn-like NSMP
Discovery Set	272	248 (91.18%)	24 (8.82%)
BC Validation Set	195	131 (67.18%)	64 (32.82%)
CC Validation Set	416	397 (95.43%)	19 (4.57%)

Supplementary Table 11: Clinicopathologic features of the *p53abn-like NSMP* group in the discovery set.

Variable	Total	NSMP	*p53abn-like NSMP*	p53abn
**Total**	363	221 (60·88%)	47 (12·95%)	95 (26·17%)
**Age at diagnosis**				
<60 yrs	121 (33·33%)	90 (40·72%)	20 (42·55%)	11 (11·58%)
≥60 yrs	242 (66·67%)	131 (59·28%)	27 (57·45%)	84 (88·42%)
**Histotype**				
Endometrioid	288 (79·34%)	220 (99·55%)	42 (89·36%)	26 (27·37%)
Non-endometrioid	75 (20·66%)	1 (0·45%)	5 (10·64%)	69 (72·63%)
**Tumour grade**				
Low grade (G1–2)	258 (71·07%)	215 (97·29%)	31 (65·96%)	12 (12·63%)
High grade (G3)	105 (28·93%)	6 (2·71%)	16 (34·04%)	83 (87·37%)
**FIGO stage**				
I-II	291 (80·17%)	203 (91·86%)	36 (76·60%)	52 (54·74%)
III-IV	71 (19·56%)	18 (8·14%)	10 (21·28%)	43 (45·26%)
Unknown	1 (0·28%)	0	1 (2·13%)	0

Supplementary Table 12: Clinicopathologic features of the *p53abn-like NSMP* group in the BC validation set without excluding any patients.

Variable	Total	NSMP	*p53abn-like NSMP*	p53abn
**Total**	288	155 (53·82%)	38 (13·19%)	95 (32·99%)
**Age at diagnosis**				
<60 yrs	81 (28·13%)	61 (39·36%)	11 (28·95%)	9 (9·47%)
≥60 yrs	205 (71·18%)	92 (59·35%)	27 (71·05%)	86 (90·53%)
Unknown	2 (0·69%)	2 (1·29%)	0	0
**Histotype**				
Endometrioid	195 (67·71%)	145 (93·55%)	27 (71·05%)	23 (24·21%)
Non-endometrioid	91 (31·60%)	8 (5·16%)	11 (28·95%)	72 (75·79%)
Unknown	2 (0·69%)	2 (1·29%)	0	0
**Tumour grade**				
Low grade (G1–2)	151 (52·43%)	140 (90·32%)	6 (15·79%)	5 (5·26%)
High grade (G3)	137 (47·57%)	15 (9·68%)	32 (84·21%)	90 (94·74%)
**FIGO stage**				
I-II	216 (75·00%)	138 (89·03%)	28 (73·68%)	50 (52·63%)
III-IV	69 (23·96%)	14 (9·03%)	10 (26·32%)	45 (47·37%)
Unknown	3 (1·04%)	3 (1·94%)	0	0

Supplementary Table 13: Clinicopathologic features of the *p53abn-like NSMP* group in the CC validation set without excluding any patients.

Variable	Total	NSMP	*p53abn-like NSMP*	p53abn
**Total**	614	390 (63.52%)	26 (4.23%)	198 (32.25%)
**Age at diagnosis**				
<60 yrs	199 (32.41%)	158 (40.51%)	10 (38.46%)	31 (15.66%)
≥60 yrs	415 (67.59%)	232 (59.49%)	16 (61.54%)	167 (84.34%)
Unknown	0	0	0	0
**Histotype**				
Endometrioid	419 (68.24%)	367 (94.1%)	13 (50.0%)	39 (19.7%)
Non-endometrioid	195 (31.76%)	23 (5.9%)	13 (50.0%)	159 (80.3%)
Unknown	0	0	0	0
**Tumour grade**				
Low grade (G1–2)	390 (63.52%)	371 (95.13%)	5 (19.23%)	14 (7.07%)
High grade (G3)	199 (32.41%)	19 (4.87%)	19 (73.08%)	161 (81.31%)
Unknown	25 (4.07%)	0	2 (7.69%)	23 (11.62%)
**FIGO stage**				
I-II	487 (79.32%)	350 (89.74%)	19 (73.08%)	118 (59.6%)
III-IV	127 (20.68%)	40 (10.26%)	7 (26.92%)	80 (40.4%)
Unknown	0	0	0	0

Supplementary Table 14: Multi-variate Cox regression analysis showing the prognostic significance of *p53abn-like NSMP* group PFS for the entire cohort.

Variable	Hazard ratio	*p*
Grade	2·35	0·04
Stage	3·94	1.2e-5
Histology (endometrioid vs. non-endometrioid)	1.1	0.82
p53abn-like NSMP	2·53	0·01
