# Supplementary material for: AI-based histopathology image analysis reveals a distinct subset of endometrial cancers
Source: Nat Commun. 2024 Jun 26;15:4973. doi: 10.1038/s41467-024-49017-2 (PMC11208496; doi:10.1038/s41467-024-49017-2)
Supplement: Supplementary file 3 — Reporting Summary [file 41467_2024_49017_MOESM3_ESM.pdf]

nature portfolio

Corresponding author(s): Ali Bashashati

Last updated by author(s): May 20, 2024

Reporting Summary

Nature Portfolio wishes to improve the reproducibility of the work that we publish. This form provides structure for consistency and transparency in reporting. For further information on Nature Portfolio policies, see our Editorial Policies and the Editorial Policy Checklist.

Statistics

For all statistical analyses, confirm that the following items are present in the figure legend, table legend, main text, or Methods section.

n/a	Confirmed	
☐	☒	The exact sample size (*n*) for each experimental group/condition, given as a discrete number and unit of measurement
☒	☐	A statement on whether measurements were taken from distinct samples or whether the same sample was measured repeatedly
☐	☒	The statistical test(s) used AND whether they are one- or two-sided<br>*Only common tests should be described solely by name; describe more complex techniques in the Methods section.*
☐	☒	A description of all covariates tested
☒	☐	A description of any assumptions or corrections, such as tests of normality and adjustment for multiple comparisons
☐	☒	A full description of the statistical parameters including central tendency (e.g. means) or other basic estimates (e.g. regression coefficient) AND variation (e.g. standard deviation) or associated estimates of uncertainty (e.g. confidence intervals)
☐	☒	For null hypothesis testing, the test statistic (e.g. *F*, *t*, *r*) with confidence intervals, effect sizes, degrees of freedom and *P* value noted<br>*Give P values as exact values whenever suitable.*
☒	☐	For Bayesian analysis, information on the choice of priors and Markov chain Monte Carlo settings
☒	☐	For hierarchical and complex designs, identification of the appropriate level for tests and full reporting of outcomes
☒	☐	Estimates of effect sizes (e.g. Cohen's *d*, Pearson's *r*), indicating how they were calculated

Our web collection on statistics for biologists contains articles on many of the points above.

Software and code

Policy information about availability of computer code

Data collection	No software was used for collecting the data.
Data analysis	The custom Python code used for data analysis, a list of all open source Python packages and their versions, installation guide, and instructions to run the code are in the attached submitted software zip folder.

For manuscripts utilizing custom algorithms or software that are central to the research but not yet described in published literature, software must be made available to editors and reviewers. We strongly encourage code deposition in a community repository (e.g. GitHub). See the Nature Portfolio guidelines for submitting code & software for further information.

Data

Policy information about availability of data

All manuscripts must include a data availability statement. This statement should provide the following information, where applicable:

- Accession codes, unique identifiers, or web links for publicly available datasets
- A description of any restrictions on data availability
- For clinical datasets or third party data, please ensure that the statement adheres to our policy

All histopathology slide images in this study can be obtained by direct email to the corresponding author. All data access are subject to institutional permission and compliance with ethics from the corresponding institutions. Data can only be shared for non-commercial academic purposes and will require a data user agreement. The whole-genome sequencing data for this study have been deposited in the European Nucleotide Archive (ENA) at EMBL-EBI under accession number

April 2023

PRJEB60600 [https://www.ebi.ac.uk/ena/browser/view/PRJEB60600]. Additionally, source data are provided with this paper. Endometrial (uterine) carcinoma samples from The Cancer Genome Atlas (TCGA), used in this study, can be freely downloaded from [https://portal.gdc.cancer.gov/analysis_page?app=Projects]. The exome-wide and targeted point mutation data discussed in this manuscript were previously published in earlier studies(4,5,64,65). The aggregated mutation calls including the genomic coordinates of the mutations and reference and tumour alleles can be found in the Source Data of Figure 3B.

Research involving human participants, their data, or biological material

Policy information about studies with human participants or human data. See also policy information about sex, gender (identity/presentation), and sexual orientation and race, ethnicity and racism.

Reporting on sex and gender	Given that the studied cancer is Endometrial cancer, which is specific to individuals with female body, gender- and sex-based analyses were not conducted. The discovery set comprised of 368 patients, whereas the validation set included 290 patients from an independent center.
Reporting on race, ethnicity, or other socially relevant groupings	NA
Population characteristics	Tables 1,2, and 3 provide the demographic and clinical features of the discovery and validation sets.
Recruitment	No patients were recruited for the study, and we conducted the designed study using the WSI images available in the three cohorts.
Ethics oversight	All study protocols have been approved by the University of British Columbia/BC Cancer Research Ethics Board.

Note that full information on the approval of the study protocol must also be provided in the manuscript.

Field-specific reporting

Please select the one below that is the best fit for your research. If you are not sure, read the appropriate sections before making your selection.

☒ Life sciences ☐ Behavioural & social sciences ☐ Ecological, evolutionary & environmental sciences

For a reference copy of the document with all sections, see nature.com/documents/nr-reporting-summary-flat.pdf

Life sciences study design

All studies must disclose on these points even when the disclosure is negative.

Sample size	Sample size calculations were not conducted, and the number of cases included in this study was determined by the availability of patients in the three cohorts. The detailed number of samples is provided in Supplementary Table 1. Furthermore, the clinicopathologic characteristics of the discovery and validation sets are described in Tables 1 and 2, respectively.
Data exclusions	Cases lacking clinical information were excluded from the study.
Replication	Following the publication of the study, the AI code implemented for this study will be made public. Furthermore, the precise hyperparameters required for reproducing the findings are delineated in the Method section. The whole-genome sequencing data will be published upon the publication of the study and histopathology slide images can be obtained by direct email to the corresponding author.
Randomization	Two cohorts were chosen as the discovery group, and one cohort was designated as the validation group. Within the discovery group, instances were randomly partitioned such that 60% were allocated to the training set, 20% to the validation set, and 20% to the testing set. Additionally, to avoid data leakage between the training, validation, and testing sets, we ensured that slides from each patient were assigned to only one of these sets.
Blinding	During the pathology review of the selected cases, each gynepathologist was blinded to the assessments of the others.

Reporting for specific materials, systems and methods

We require information from authors about some types of materials, experimental systems and methods used in many studies. Here, indicate whether each material, system or method listed is relevant to your study. If you are not sure if a list item applies to your research, read the appropriate section before selecting a response.

Materials & experimental systems

n/a	Involved in the study
☒	☐ Antibodies
☒	☐ Eukaryotic cell lines
☒	☐ Palaeontology and archaeology
☒	☐ Animals and other organisms
☒	☐ Clinical data
☒	☐ Dual use research of concern
☒	☐ Plants

Methods

n/a	Involved in the study
☒	☐ ChIP-seq
☒	☐ Flow cytometry
☒	☐ MRI-based neuroimaging
